# CpsR, a GntR family regulator, transcriptionally regulates capsular polysaccharide biosynthesis and governs bacterial virulence in *Streptococcus pneumoniae*

**DOI:** 10.1038/srep29255

**Published:** 2016-07-08

**Authors:** Kaifeng Wu, Hongmei Xu, Yuqiang Zheng, Libin Wang, Xuemei Zhang, Yibing Yin

**Affiliations:** 1 Key Laboratory of Diagnostic Medicine designated by the Ministry of Education, Chongqing Medical University, Chongqing 400016, People’s republic of China

## Abstract

Transcriptional regulation of capsule expression is critical for pneumococcal transition from carriage to infection, yet the underlying mechanism remains incompletely understood. Here, we describe the regulation of capsular polysaccharide, one of the most important pneumococcal virulence factor by a GntR family regulator, CpsR. Electrophoretic mobility-shift assays have shown the direct interaction between CpsR and the *cps* promoter (*cpsp*), and their interaction could be competitively interfered by glucose. DNase I footprinting assays localized the binding site to a region −146 to −114 base pairs relative to the transcriptional start site of the *cps* locus in *S. pneumoniae* D39. We found that CpsR negatively controlled the transcription of the *cps* locus and hence CPS production, which was confirmed by fine-tuning expression of CpsR in a Δ*cpsR* complemented strain. Increased expression of CpsR in complemented strain led to a decreased resistance to the whole-blood-mediated killing, suggesting a protective role for CpsR-*cpsp* interaction in the establishment of invasive infection. Finally, animal experiments showed that CpsR-*cpsp* interaction was necessary for both pneumococcal colonization and invasive infection. Taken together, our results provide a thorough insight into the regulation of capsule production mediated by CpsR and its important roles in pneumococcal pathogenesis.

*Streptococcus pneumoniae* (pneumococcus) is known as an important human bacterial pathogen, which usually resides in the upper respiratory tract and has the capacity to cause pneumonia, otitis media, meningitis and sepsis^1^. It has been estimated in 2010 that at least 400,000 children under 6 years of age die each year from invasive pneumococcal disease worldwide^2^.

An infection generally occurs when host factors are in favor of bacterial growth^3^ and allow a pathogen to modulate the expression of virulence factors to survive a host’s defense mechanism^4^,^5^. The pneumococcus harbors a number of virulence factors, including capsular polysaccharide, pneumolysin, pneumococcal surface antigen A, choline binding proteins^6^. Of them, the capsular polysaccharide (capsule, CPS) is recognized as the most important one contributing to the disease process^6^. CPS has several distinguishing features^7^,^8^,^9^,^10^, one of the most striking being the role of its protection against host opsonophagocytic killing^11^. Despite CPS contributes to both pneumococcal colonization and invasive disease^12^, different lines of evidence indicate that CPS is differentially produced between carriage pneumococci and blood-grown planktonic pneumococci^13^,^14^,^15^,^16^; nevertheless, the underlying mechanism is not yet well understood.

It’s accepted that transcript levels of the *cps* locus are positively correlated with the amount of CPS^17^, and *cps2A* gene has been normally used to reflect the transcription of the *cps* locus in *S. pneumoniae*^18^. Although different *S. pneumoniae* serotypes varied in the ability to colonize the nasopharynx or cause invasive infection, estimation of the level of *cps2A* transcript produced by 14 different serotypes showed that strains with high carriage potential (6A, 6B, 9V, 15, 18C, 19F, 23F, and 33) expressed an average of two-fold more *cps2A* than the invasive serotypes (1, 4, 5, 7F, 8 and 14)^19^. In addition, a detailed comparison of the strain cultured *in vitro* or isolated from the blood has revealed that transcript level of the *cps2A* gene is linked to the survival of pneumococci in the blood^20^. Thus, transcriptional regulation of CPS production in response to surrounding stimuli is probably a principal mechanism responsible for pneumococcal pathogenesis.

With the exception of serotype 37, the biosynthesis of CPS is closely dependent on the *cps* locus which is located between the *dexB* and *aliA* genes in *S. pneumoniae*^21^,^22^. Sequences of the *cps* locus promoter (*cpsp*) and the first four genes are highly conserved among pneumococcal strains^23^,^24^. In contrast, *cps2D* downstream genes are serotype-specific, which are involved in either polymerization or export of capsular polysaccharide^24^. By searching the *cps* promoter-proximal region investigators have revealed some motifs with homology to the binding sites of transcription regulators including RegM, CcpA and GlnR; however, there is no experimental evidence indicating the direct regulation between the regulators and the *cps* locus^25^. In addition, Spd0729 was reported to transcriptionally regulate the expression of the *cps* locus, but the underlying mechanism was not investigated in their work^18^. Two recent studies have demonstrated the essential roles for the spacing sequence and the core promoter region in transcriptional regulation of the *cps* locus; specifically, unmarked deletion of spacing sequence or core promoter region in the promoter region led to a significantly reduced *cps* transcription and hence reduced CPS production^17^,^26^. Despite of these observations, transcription factors that can directly bind the *cpsp* and control CPS production remain uncharacterized, and the role of transcription regulators in pneumococcal pathogenesis remains incompletely understood.

To gain insights into the transcriptional regulation of the *cps* locus, 5′-biotin labeled *cpsp* DNA was used to fish out potential transcriptional regulators using DNA affinity chromatography, which led to identification of six potential regulators. In this study, we have performed in-depth molecular analyses of SPD_0064 (referred to as CpsR, the *cps* locus repressor), and show that CpsR binds directly to the *cpsp* and hence negatively regulates CPS production. More importantly, we found that the interaction of CpsR with *cpsp* is further controlled by glucose, which may be one of the mechanisms accounting for the transition from carriage to invasive disease in *S. pneumoniae*. Fine-tuning expression of CpsR in a Δ*cpsR*-C strain was used to reveal the impact of CpsR-mediated CPS production on resistance to whole-blood-mediated killing. Finally, we provide evidence that CpsR is required for the pneumococcal nasopharyngeal colonization and systemic infection in mice.

## Results

### Discovery of the transcriptional regulators that bound to the upstream region of the *cps* locus

**Figure 1:**
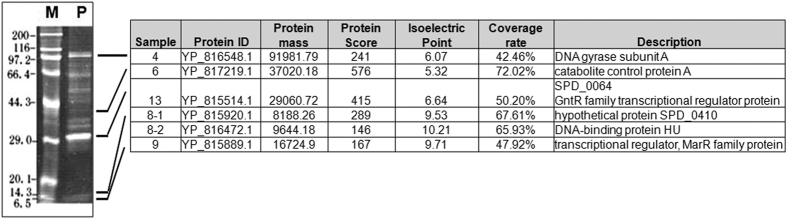
SDS-PAGE and MS results of *S. pneumoniae* D39 proteins fished out by affinity chromatography using the *cpsp* DNA as bait. Enriched proteins were excised from the SDS-PAGE gel and identified by MALDI-TOF mass spectrometry. Lane M indicates molecular mass markers. Lane P indicates that proteins were eluted with 0.3 M NaCl solution. The numbers on the left indicate the molecular weights of the proteins.

It’s known that the transcription of *cps* genes is closely dependent on the activity of the *cps* promoter (*cpsp*). Wen *et al*. has recently updated the information regarding the transcription start site (TSS) of the *cps* locus in *S. pneumoniae* strain D39, which is located 25 bp upstream from the first nucleotide of the *cps2A* translation initiation codon^17^. By searching the promoter-proximal sequence, a previous bioinformatic analysis suggested the existence of several operator sequences similar to the binding sites including of CCPA, CodY, GlnR, and RitR^25^. However, whether these regulators are involved in transcriptional activation of the *cps* locus is still lack of experimental evidence. Previous studies demonstrated that the core promoter region, spacing sequence (SS) and repeat sequence of pneumococcus (RUP) are required for the transcriptional regulation of the *cps* locus^17^,^26^. Based on these findings, a 5′-end biotin-labeled *cpsp* DNA (218 bp in length, from −177 to +41 nt relative to the transcriptional start site) was used to search for the potential regulators of the *cps* locus using DNA affinity chromatography. DNA-binding proteins were visualized by SDS-PAGE and identified by MALDI-TOF mass spectrometry after tryptic digestion. This screening led to the discovery of six candidate regulators including CcpA, a GntR family transcriptional regulator SPD_0064, a MarR family transcriptional regulator SPD_0379, DNA binding protein HU and DNA gyrase subunit A (Fig. 1). Of these proteins, SPD_0064 was of particular interest because it belongs to the GntR family transcription regulator (Fig. S1) and may have the capacity to bind glucose^27^. Examination of the localization of *SPD_0064* gene revealed that it is located approximately 25,000 nt apart from the *cps* locus in *S. pneumoniae* D39. Because SPD_0064 negatively controls the transcription of the *cps* locus, it was then designated as CpsR (for the *cps* locus repressor, and hence the *cpsR* gene). The sequence of CpsR consists of 247 amino acids, and the protein has a calculated mass weight of 28.9 kDa. Like other GntR family regulators, CpsR has two typical domains, including a highly conserved N-terminal helix-turn-helix DNA-binding domain (amino acid residues 13 to 78, Fig. S1A) and a C-terminal regulatory ligand-binding domain (amino acid residues 97 to 237, Fig. S1B). In the following sections, the role and underlying mechanisms of CpsR in *cps* transcription and pneumococcal pathogenesis are described.

### Binding of GntR to the *cps* locus promoter region

**Figure 2:**
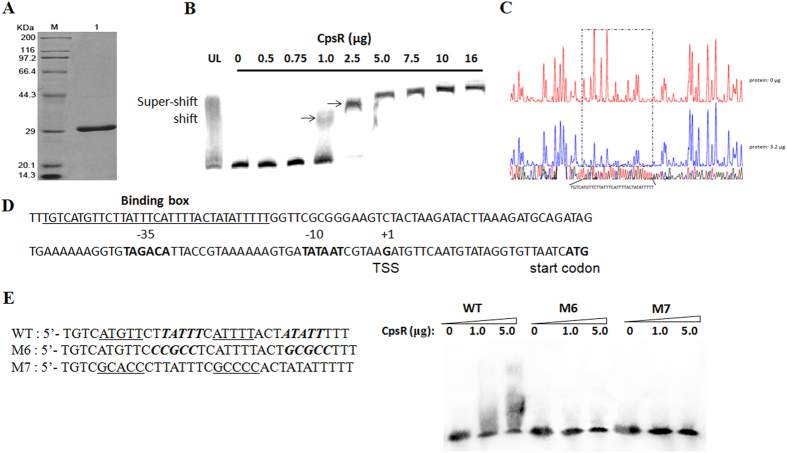
Characterization of the interaction between CpsR and the *cpsp*. (**A**) The purity of 6His-CpsR protein analyzed with SDS-PAGE. The molecular weight of 6His-CpsR is about 29 kDa. M, mass makers; lane 1, purified 6His-CpsR protein. (**B**) EMSA of 6His-CpsR protein. The biotin-labeled 218-bp *cpsp* probe (8 fmol) was incubated with the indicated amounts of 6His-CpsR protein. For the competition analysis, the same but unlabeled (UL) DNA probe was included as 400-fold concentration relative to the biotin-labeled probe. (**C**) DNase I footprinting protection assay of CpsR. The addition of 3.2 μg 6His-CpsR protected a 33-bp fragment from digestion by DNase I. (**D**) Structural organization of the *cps* promoter-proximal region. The CpsR-protected 33-bp DNA sequence is underlined and named binding box, the putative −10 and −35 elements, transcriptional start site (TSS), start codon are in bold. (**E**) Mutated sequences are either underlined (M7) or in italic (M6). EMSA of 6His-CpsR with wild-type probe (WT) or mutants of the probe (M6 and M7), different amounts of 6His-CpsR protein were incubated with 0.25 μM of the wild-type or mutated probes.

Because CpsR was obtained from the DNA affinity chromatography screening, we hypothesized that CpsR could bind directly to the *cpsp* DNA fragment. To this end, electrophoretic mobility-shift assays (EMSAs) were carried out. 6His-CpsR protein was produced in *E. coli* BL21 (DE3) and purified by Ni-NTA affinity chromatography (Fig. 2A). Next, purified 6His-CpsR protein was incubated with the 5′-biotin labeled *cpsp* probe used in the DNA affinity chromatography screening. Gel shift assays demonstrated that purified 6His-CpsR could bind to the *cpsp*, showing a dose-dependent pattern (Fig. 2B). Shifted bands were observed when over 1.0 μg of CpsR was added to the reaction mixture. Super-shifted bands were observed at higher CpsR concentrations, which may reflect the formation of dimmers or tetramers of the protein. The incubation of the *cpsp* DNA fragment with 10 μg of nonspecific protein LytR or nonspecific DNA with CpsR did not result in band shifting (Fig. S2). The mobility shift could be inhibited by coincubation of biotin-labeled *cpsp* with CpsR and a 400-fold excess of unlabeled *cpsp* DNA (Fig. 2B), suggesting a specific shift for CpsR. These data provide strong evidence for the binding of CpsR to the *cpsp*.

To investigate further the mechanism of CpsR in regulation of the *cps* locus, we searched for potential CpsR-binding sites in the promoter region of the *cps* locus using a dye-based DNase I footprinting analysis. As shown in Fig. 2C, in the presence of 6His-CpsR, an apparent region of 33 nt of the *cps* promoter, 5′-TGTCATGTTCTTATTTCATTTTACTATATTTTT-3′, was protected against DNase I digestion, spanning from nucleotide −146 to −114 relative to the TSS of the *cps* locus in *S. pneumoniae* D39 (Fig. 2D). To confirm binding of CpsR to the binding site obtained from DNase I footprinting assays, we mutated the CpsR-protected region with the indicated nucleotides. Wild-type and mutated probes were synthesized and 5′-biotin labeled for EMSA with purified 6His-tagged CpsR. As shown in Fig. 2E, wild-type probe was obviously retarded; whereas the mutated fragments showed less interaction, particularly with the M7 probe. These findings indicate that the binding site is crucial for the specific interaction between CpsR and the promoter of the *cps* locus.

### Effect of *cpsR* gene deletion on transcription of the *cps* locus and CPS production

**Figure 3:**
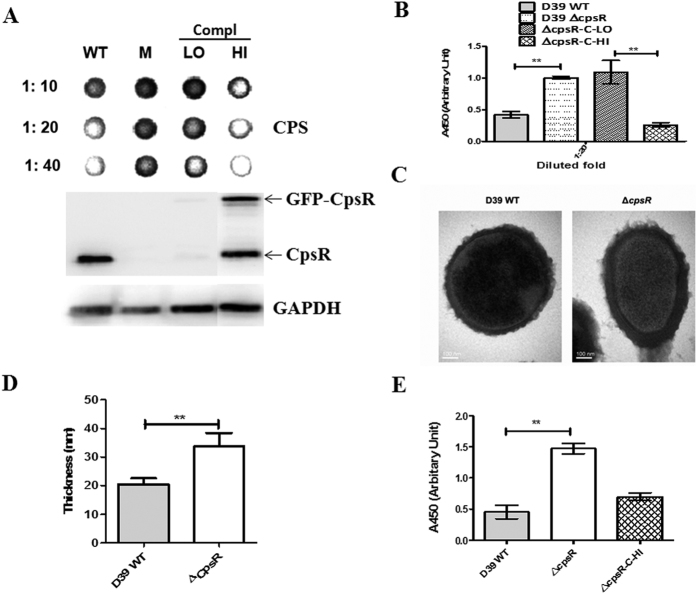
CpsR negatively regulates CPS production. (**A**) Detection of CPS production in D39 and its derivatives by dot blotting. A representative dot blot image is shown. WT: wild-type D39 strain; M: D39Δ*cpsR*; Compl: complemented strain. HI: complemented cells were grown in medium supplemented with 0.15 μM ZnCl_2_; LO: complemented cells were grown in normal C+Y medium. Proteins in lysate of D39, Δ*cpsR*, and Compl were probed with either polyclonal murine anti-CpsR or anti-GADPH serum. CPS was probed with rabbit anti-serotype 2 serum. The numbers on the left side indicate the dilutions of the sample. (**B**) Quantitative CPS levels were determined by ELISA. Bacteria were lysed by 0.5% DOC solution and then 1: 20 diluted by PBS before being subjected to ELISA assays. Results are mean (SD) from three independent experiments. **P < 0.01. (**C**) Representative TEM images of wild-type D39 and ΔcpsR mutant. The bar (magnification is 120,000) indicates 100 nm. (**D**) Capsule thickness (nm) measured with ImageJ software. The data are expressed as means ± S.D. **P < 0.01. Statistical difference was determined by unpaired two-tailed Student’s *t* test. (**E**) Quantitative ELISA analysis of CPS levels in the supernatants of cultures of bacteria. Results are mean (SD) from three independent experiments. **P < 0.01. Statistical difference was determined by unpaired two-tailed Student’s *t* test.

To gain insight into the function of *cpsR*, we constructed D39Δ*cpsR* mutant using insertion-deletion mutagenesis strategy. The *cpsR* gene was deleted from the chromosome of *S. pneumoniae* by *erm* cassette replacement, which was a nonpolar event verified by reverse transcription (RT)-PCR. An ectopically complemented strain (Δ*cpsR*-C) was constructed by integrating the pJWV25-*cpsR* plasmid *via* double cross-over into the *bgaA* locus in D39Δ*cpsR* strain. This resulted in GFP + -CpsR expression under the control of the Zn^2+^. Sequencing of the respective genome regions confirmed that the mutations or insertions generated as expected. Immunoblots with murine polyclonal CpsR antiserum revealed the production of CpsR in wild-type D39 strain, and the absence of CpsR in D39Δ*cpsR* (Fig. 3A). As expected, the expression of CpsR could be well fine-tuned by the addition of Zn^2+^ in growth medium of the Δ*cpsR*-C strain (Fig. 3A).

Immnoblotting and ELISA assays were performed to determine the production of CPS in lysates of D39, D39Δ*cpsR*, and Δ*cpsR*-C. Wild-type D39 and D39Δ*cpsR* strains were grown in normal C+Y medium. For Δ*cpsR*-C strain, cells were grown in either normal C+Y medium (8 μM ZnCl_2_) to have a phenotype of CpsR^LOW^CPS^HIGH^ (Δ*cpsR*-C-LO) or C+Y medium supplemented with high concentration of ZnCl_2_ (0.15 mM) to have a phenotype of CpsR^HIGH^CPS^LOW^ (Δ*cpsR*-C-HI). Strains were grown to identical culture densities of OD_600_ = 0.4. Bacteria were harvested, re-suspended in lysis buffer, and then subjected to immunoblotting assays using rabbit anti-serotype 2 serum and Western blotting using polyclonal mouse anti-CpsR sera. GADPH was used as an internal control. Dot blot results showed an increased production of CPS in D39Δ*cpsR* mutant compared to the wild-type D39 strain (Fig. 3A). Compared to Δ*cpsR*-C cells grown in normal C+Y medium, Δ*cpsR*-C cells grown in C+Y medium supplemented with 0.15 mM ZnCl_2_ produced an increased expression of CpsR but a reduced amount of CPS (Fig. 3A). To confirm the immunoblot results, we directly quantified the amount of CPS of the strains by ELISA. We observed a significantly increased CPS production for D39Δ*cpsR* mutant than the wild-type D39 strain (Fig. 3B). Also, Δ*cpsR*-C-LO cells produced significantly more CPS than Δ*cpsR*-C-HI cells, indicating a reverse relationship between CPS production and CpsR expression.

We further examined the capsule thickness of wild-type D39 strain and Δ*cpsR* mutant using transmission electron microscopy. The result demonstrated that the capsules of the ∆*cpsR* mutant were thicker than those of wild-type strain (Fig. 3C). The mean capsule thickness for wild-type D39 strain and D39∆*cpsR* mutant were 20.5 nm and 33.75 nm respectively (Fig. 3D). The statistical result showed that the average capsule thickness of the ∆*cpsR* strain was significantly increased compared with that of the wild-type strain (Fig. 3E).

To explore whether the effect of CpsR on *cps* gene expression was influenced by host strain, *cpsR* deletion mutant was constructed in a serotype 19F strain (CMCC(B)31693). The 19FΔ*cpsR* mutant also exhibited an increased CPS expression relative to the wild-type 19F strain, as judged by immunoblotting and ELISA assays (Fig. S3). To rule out the possibility that the reduced CPS production is caused by an impaired anchorage of CPS to the cell surface, the amount of CPS in the culture supernatants were tested. The result showed a similar increased CPS in the culture supernatant of the D39Δ*cpsR* strain (Fig. 3E), confirming that CpsR could negatively control CPS production.

**Figure 4:**
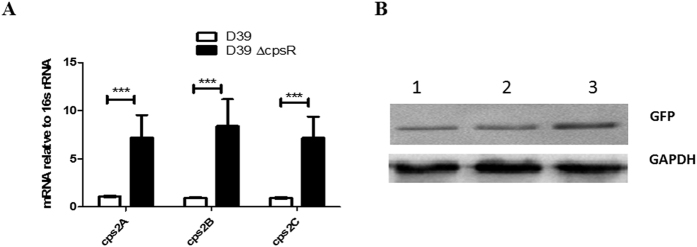
Effect of the *cpsR* deletion or *cpsp* mutations on *cps* transcription in *S. pneumoniae* D39. (**A**) Real time RT-PCR analysis of *cps2A-C* mRNA. mRNA levels are expressed relative to 16sRNA. Data represents the mean (SD) from three independent experiments each determined in duplicate. ***P < 0.001, analyzed by unpaired two-tailed Student’s *t* test. (**B**) The promoter activities of wild-type and mutated *cps* promoters in *S. pneumoniae* D39 background. The expression of GFP, driven by the wild-type *cpsp* (lane 1) or mutated promoters, *cpsp*^M6^ (lane 2) or *cpsp*^M7^ (lane 3), was determined by Western blot with monoclonal anti-GFP antibody, and the expression of GADPH was served as loading control.

To determine whether the expression of the *cps* locus is transcriptionally regulated by CpsR, qRT-PCR was performed to determine the relative levels of *cps2A-C* mRNA in cultures of wild-type D39, D39Δ*cpsR* grown in C+Y to an OD_600_ of 0.4. The 16S rRNA levels were used as an internal control. Real time RT-PCR was performed in duplicate on three independently isolated mRNA samples for each strain. In D39Δ*cpsR*, the *cps2A/B/C* mRNA levels were up-regulated approximate 6- to 8-fold relative to those in wild-type D39 (*P* < 0.001, Fig. 4A).

To address whether the binding site was also important for the transcriptional activity, we constructed transcriptional reporter plasmids by fusing the wild-type promoter (*cpsp*^WT^) and the mutated promoters (*cpsp*^M6^ and *cpsp*^M7^) to the reporter gene *gfp* respectively. Plasmids were integrated into the *cps* locus of *S. pneumoniae* D39 strain via single crossover recombination. Western blotting analyses of GFP were conducted to reflect the activities of the promoters. Immunoblots with polyclonal GFP antibody showed that the expression of GFP was enhanced under the mutated promoters than the wild-type promoter, particularly with the *cpsp*^M7^ (Fig. 4B). These results indicate that CpsR could directly bind to the promoter region of the *cps* locus and the 33 nt sequence is essential for the binding of CpsR with the *cpsp*.

Together these data indicate that the transcription of the *cps* locus is negatively and directly regulated by the CpsR in *S. pneumoniae*.

### CpsR is a glucose sensing regulator

**Figure 5:**
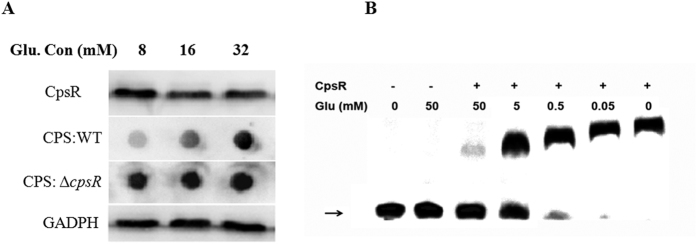
CpsR is a glucose sensing regulator. (**A**) wild-type D39 strain and D39Δ*cpsR* mutant were grown in C+Y media supplemented with the indicated concentrations of glucose. Bacteria were collected for Western blots of CpsR and dot blots of CPS production. CpsR and GADPH were probed with murine anti-CpsR or anti-GADPH serum, respectively. CPS was probed with rabbit anti-serotype 2 serum. (**B**) EMSA of CpsR-*cpsp* complex in the presence of the indicated concentrations of glucose. Unbound DNA is indicated with an arrow.

To test whether glucose could be a signal trigger in controlling *cps* transcription, wild-type D39 strain was grown in C+Y medium supplemented with different concentrations of glucose. The result showed that CPS production of the wild-type D39 strain was increased with the increasing of glucose concentrations. In contrast, alternation in CPS production was not observed for D39Δ*cpsR* mutant grown in media supplemented with concentrations of glucose tested in this study (Fig. 5A). As can be seen in Fig. 5A, the addition of glucose has a relatively little influence on the expression of CpsR. To further explore molecular mechanisms associated with glucose in regulation of CPS expression, we tested whether the addition of glucose influences the binding of CpsR with its operator using gel shift assays. A serially 10-fold-increased concentration of glucose was added into the EMSA reaction mixture. Gel shift assays showed evident liberation of the *cpsp* DNA from the CpsR-*cpsp* complex at glucose concentrations above 0.5 mM (Fig. 5B). These *in vitro* data indicate that the glucose can control CPS production, mainly by interfering with the binding of CpsR with the *cpsp*.

### Role of CpsR in resistance to the whole-blood killing

**Figure 6:**
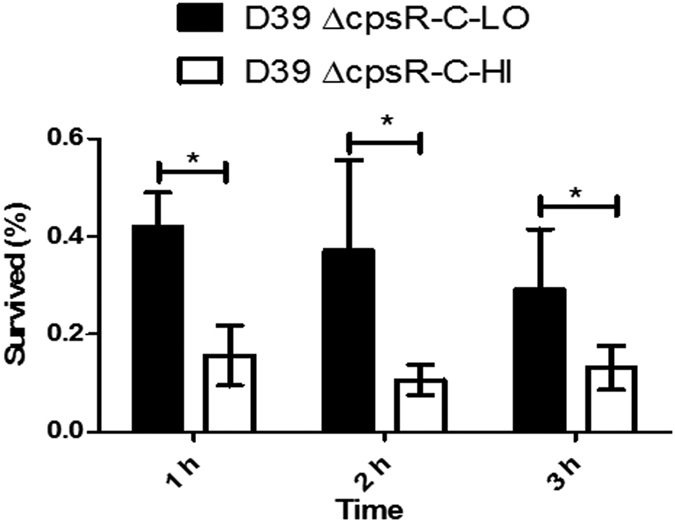
Overexpression of CpsR caused decreased resistance to whole-blood-mediated killing. Survival for Δ*cpsR*-C-LO cells (a CpsR^LOW^-CPS^HIGH^ phenotype) or Δ*cpsR*-C-HI cells (a CpsR^HIGH^-CPS^LOW^ phenotype) following incubation with mouse whole blood. Bacteria (~120 CFU) in 10 μl were added to mouse heparinized whole blood (90 μl), then mixed and incubated for 1, 2, or 3 h at 37 °C. Following incubation, the number of CFU was determined. Data represents the mean (SD) (n = 5). *P < 0.05. Statistical difference was determined by unpaired two-tailed Student’s *t* test.

Given the apparent role of CpsR in regulation of CPS production, we next determined the contribution of CpsR to pneumococcal survival with mouse whole blood. For comparative purposes, the experiment was undertaken with Δ*cpsR*-C cells whose CPS production could be controlled by Zn^2+^ in the culture medium. Δ*cpsR*-C cells were grown in normal C+Y medium (referred to as Δ*cpsR*-C-LO, a CpsR^LOW^-CPS^HIGH^ phenotype) or C+Y medium supplemented with 0.15 mM ZnCl_2_ (referred to as Δ*cpsR*-C-HI, a CpsR^HIGH^-CPS^LOW^ phenotype) to generate cells with apparent different expression of CpsR and CPS in the same genetic background. Then, bacterial cells (~120 CFU) in 10 μl were incubated with 90 μl mouse heparinized whole blood and survival rates of the strains were determined. As shown in Fig. 6, 41.8%, 37.1% and 29.1% of Δ*cpsR*-C-LO cells survived from killing by mouse blood following 1-, 2- and 3-h incubation, respectively. On the contrary, only 15.6%, 10.6% and 13.1% of Δ*cpsR*-C-HI cells survived from the killing following 1-, 2- and 3-h incubation, respectively. Thus, overall, there was approximately 2- to 3-fold reduction in survival of the Δ*cpsR*-C-HI variant compared to the survival of the Δ*cpsR*-C-LO variant. These results indicate that down-regulation of CpsR increases the pneumococcal resistance to whole-blood-mediated killing.

### The role of CpsR in systemic virulence and nasopharyngeal colonization

**Figure 7:**
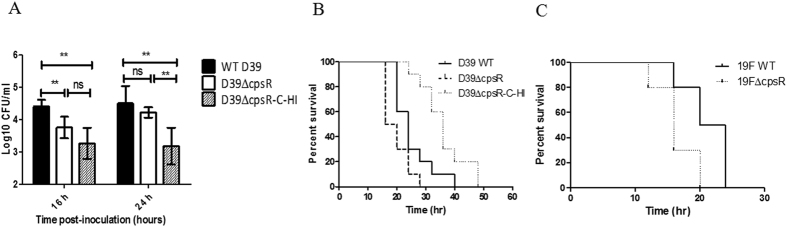
The role of CpsR in nasopharyngeal colonization and systemic virulence. (**A**) Mice were challenged with 10^6^ CFU and pneumococci were recovered from the nasopharynx 16 or 24 h after intranasal infection of Balb/c mice. The graphs show log_10_ CFUs (SD) recovered from nasopharynx of five Balb/c mice for D39, Δ*cpsR* or Δ*cpsR*-C-HI (a CpsR^HIGH^-CPS^LOW^ phenotype). Data are the mean (SD). **P < 0.01; *P < 0.05; ns, not significant. Statistical difference was determined by unpaired two-tailed Student’s *t* test. Balb/c mice were infected intraperitoneally with 10^3^ cells of *S. pneumoniae* D39 or their derivates (**B**) or 10^7^ cells of a serotype 19F strain or the 19FΔ*cpsR* mutant (**C**), respectively. Survival times were recorded. D39 WT *vs* Δ*cpsR*, *P* = 0.029; D39 WT *vs* ΔcpsR-C-HI (a CpsR^HIGH^-CPS^LOW^ phenotype), *P* = 0.0037; Δ*cpsR vs* ΔcpsR-C-HI (a CpsR^HIGH^-CPS^LOW^ phenotype), *P* < 0.0001. 19F WT *vs* 19FΔ*cpsR, P* = 0.0057. Statistical difference was analyzed by the log-rank (Mantel-Cox) test.

As mentioned above, the down-regulation of *cpsR* resulted in increased CPS production which confers resistance to whole-blood-mediated killing. Therefore we sought to assess whether CpsR expression has impact on pneumococcal colonization and virulence. To determine whether CpsR is required by *S. pneumoniae* to colonization, groups of 10 Balb/c mice were inoculated intranasally with wild-type D39, D39Δ*cpsR*, or Δ*cpsR*-C-HI (a CpsR^HIGH^-CPS^LOW^ phenotype) at a dose of 10^6^ CFU. Five mice from each group were killed after 16 and 24 h, and the numbers of bacteria recovered from nasopharynx were determined and compared. Compared to wild-type D39 strain, Δ*cpsR* mutant showed a significant decrease in numbers of pneumococci colonizing the nasopharynx at 16 h (two-tailed Student’s *t* test, *P* < 0.01; Fig. 7A). One day after the inoculation, Δ*cpsR* mutant was also reduced in colonization compared to wild-type strain, despite not showing any statistically significant difference. Furthermore, a significant decrease in bacterial load of the nasopharynx was observed at both 16 h and 24 h postinoculation with the Δ*cpsR*-C-HI variant compared with the wild-type D39 strain. These results suggest that CpsR-regulated encapsulation is important for pneumococcal colonization of the nasopharynx in mice.

To investigate the role of CpsR in systemic infection, groups of mice were challenged intraperitoneally with wild-type D39, D39Δ*cpsR*, or Δ*cpsR*-C-HI (a CpsR^HIGH^-CPS^LOW^ phenotype) at a dose of 10^3^ CFU. As shown in Fig. 7B, although mice were eventually die from infection, mice infected with wild-type D39 strain survived much longer than those infected with D39Δ*cpsR* mutant (Log-rank test, *P* = 0.029). Δ*cpsR*-C-HI was less virulent compared to the wild-type D39 strain (Log-rank test, *P* = 0.0037). In addition, virulence potential was evaluated using a serotype 19F strain (CMCC(B)31693) and 19FΔ*cpsR* mutant. As expected, 19FΔ*cpsR* mutant was much more virulent than wild-type 19F strain in invasive disease (Fig. 7C; Log-rank test, *P* = 0.0057). Overall, consistent with the results obtained from whole-blood-mediated killing assays, the results demonstrate that the capsule increases pneumococcal virulence in the mouse sepsis model.

## Discussion

Capsular polysaccharide plays important roles in *S. pneumoniae* disease processes. Transcriptional regulation of the *cps* locus is recognized to be an efficient way to modulate pneumococcal CPS production^17^,^26^. However, regulators involved in the direct regulation of *cps* transcription have not been identified. In the present study, we report for the first time that *cps* transcription is negatively and directly regulated by a GntR family regulator, CpsR. Furthermore, our results show that glucose may act as a signal trigger from the extracellular milieu to activate *cps* gene expression, which may be a crucial aspect of invasive pneumococcal disease. Finally, we provide evidence that CpsR-regulated CPS production is important for both pneumococcal colonization and invasive disease.

In the present study, DNA affinity chromatography and MALDI-TOF mass spectrometry were used to search for and identify possible transcriptional regulators of the *cps* locus. This is a well established method to fish for regulators that potentially involve in regulation of the target genes^27^,^28^. The present work further confirms the efficiency of this strategy by showing the identification of six possible regulators for the *cps* locus.

The GntR family has a large number of transcriptional regulators that contain a conserved N-terminal helix-turn-helix DNA-binding domain and a C-terminal ligand regulatory domain. For example, *V. cholerae* FadR, a GntR family member, has typical N-terminal DNA binding domain and a C-terminal ligand regulatory domain^29^. Like *V. cholerae* FadR, *S. pneumoniae* CpsR has the similar general domains. By sequence alignment using Clustal W method, we observed that amino acid sequences of CpsR spanning residues Y17 to F76 showed 33.3% identity to the region of *V. cholerae* FadR (Y15 to F74), suggesting that *S. pneumoniae* CpsR may also bind to the specific binding site as a homodimer. This notion was supported by gel shift results showing the existence of super shift bands (Fig. 2B). A typical mirror repeat, 5′-TTATTTCATT*TTACTATATT*-3′, was found in the binding site, which may provide binding sequence basis for CpsR to be a homodimer after binding to its operator. Nevertheless, structural study of CpsR and its complexes with the operator need to be further investigated to address this hypothesis.

The upstream sequence of the *cps2A* start codon (promoter-proximal region) is generally composed of some insertion elements, a repeat unit of pneumococcus (RUP), the spacing sequence (SS) and the core promoter (−10 and −35 boxes) in the majority of the pneumococcal strains^17^. Work by Shainheit *et al*. has revealed the essential role of the core promoter of the *cps* operon (between the −35 box and the transcription start site of the *cps* operon) in transcriptional regulation of capsule level^26^. Wen *et al*. confirmed their results and revealed further the requirement of the SS sequence in transcription regulation of capsule locus^17^. In this work, we also show the importance of the region spanning RUP and SS sequences in transcription regulation of the *cps* locus in *S. pneumoniae*, extending our knowledge on the importance of the upstream sequence in regulation of capsule biosynthesis.

It is clear that transcriptional regulation has a major influence on adaption of *S. pneumoniae* to changing host environments during infection. Several studies have demonstrated the changes in the expression of virulence factors following the invasive disease^14^,^16^,^30^. The molecular mechanisms underlying *S. pneumoniae* carriage and virulence remain largely incomplete. A better understanding of how this pathogen controls its gene expression, particularly those having critical roles in invasive disease, is essential to design more effective treatment and prophylaxis strategies against pneumococcal infection. Previous studies have demonstrated the contribution of transcriptional regulators in virulence but were partially characterized^5^,^31^. For example, *S. pneumoniae* two-component signal transduction systems have been reported to play a role in the ability of *S. pneumoniae* to adapt to changing environments^5^. The current study extends our understanding on the regulation mechanism of pneumococcal pathogenesis showing that CpsR is required for the transcription of the *cps* locus and CPS production.

Like GntR family transcription factor PckR, *S. pneumoniae* CpsR also has an UbiC transcription regulator-associated domain in its C-terminal region, and this domain was proposed to have the capacity to bind small molecules including glucose^27^. It has been reported in *V. cholerae* that, in the presence of long chain fatty acyl-CoA, FadR will activate the *plsB* transcription by detaching itself from its operator sites^29^,^32^. Thus, we hypothesized that glucose may work in the same way. To this end, we incubated CpsR with the *cpsp* in the presence of glucose and gel shift assays were used to show the role of glucose on CpsR-*cpsp* binding. We found that the *cpsp*-binding activity of CpsR was influenced by glucose in a dose dependent pattern, suggesting that glucose may interact with CpsR to control its repressor activity.

Recent clinical and epidemiological data suggest respiratory virus infections are important risk factors for acquisition of pneumococcal disease^14^. Host immune modulation following the viral infection has been shown to contribute to secondary pneumococcal infection^33^,^34^, but investigation was seldom performed from the perspective of pathogen itself. Recently, work by Siegel and coworkers has shown that influenza-induced release of sialic acid contributes to pneumococcal growth and colonization, underscoring the importance of nutrient substances in the following pneumococcal infection^35^. Glucose is another important nutrient factor, which is not normally present in airways secretions but appears in patients with hyperglycemia or having viral infection who were at high risk of developing pneumonia^36^,^37^,^38^,^39^. It has been reported that glucose can augment bacterial growth by acting as an energy source in the respiratory tracts^3^,^40^; however, this alone seems not enough for colonizing pneumococci to cause invasive infection, since Sanchez *et al*. demonstrated that pneumococci in biofilm were not able to cause invasive disease^15^. We therefore hypothesized that glucose may serve as a host signal to trigger pneumococci modulating virulence gene expression and hence adapting to different microniches. In the present study, we show that the amount of CPS was increased with increasing of glucose concentrations in wild-type D39 strain (Fig. 5A), suggesting glucose is a positive regulator for CPS biosynthesis in *S. pneumoniae*. This was confirmed by band shift assays showing that the interaction of CpsR-*cpsp* could be interfered with the addition of glucose (Fig. 5B). These findings implicate the important carbon source, glucose, in regulating the pneumococcal virulence by controlling CpsR-mediated *cps* gene expression. In addition, our findings may be used to explain why *S. pneumoniae* *cps* gene can be differentially expressed in changing *in vitro* and *in vivo* environments.

It is of note that despite we named Δ*cpsR*-C as the complemented strain, as shown in Fig. 3A, the phenotype of Δ*cpsR*-C variant was determined by the Zn^2+^ concentration in growth conditions. Δ*cpsR*-C-HI variant produced less capsule than the wild-type D39 strain before challenge of mice and the concentrations of Zn^2+^ may not be optimal to sustain *cps* expression after inoculation, thus, the phenotype of the complemented strain (Δ*cpsR*-C-HI) does not fully reflect that of the wild-type D39 strain (Fig. 7A,B). In the present study, Δ*cpsR*-C-HI variant colonized the nasopharynx at a significant lower level than the wild-type D39 strain. This might be unexpected because a reduced capsule should facilitate interaction between pneumococci and host cells, and therefore increased numbers should be present in the nasopharynx of mice. However, this observation may be at least partially explained by the impairment of Δ*cpsR*-C-HI cells in resistance to host killing (Fig. 6). In addition, our results show that Δ*cpsR* mutants were reduced in their ability to colonize the nasopharynx in comparison with the wild-type parents. One possible explanation could be that thick capsule may preclude the interaction between pneumococci and cells^41^. In consistent with the *in vitro* result that increased CPS production is linked to a greater resistance to whole-blood-mediated killing (Fig. 6), Δ*cpsR* mutants are increased in virulence in systemic infection models (Fig. 7B,C). In this sense, upregulation of CPS production seems to be advantageous for invasive pneumococcal infection because of its protective effect against host killing. Taken together, our data suggest the importance of CpsR-regulated encapsulation in both colonization and invasive infection.

**Figure 8:**
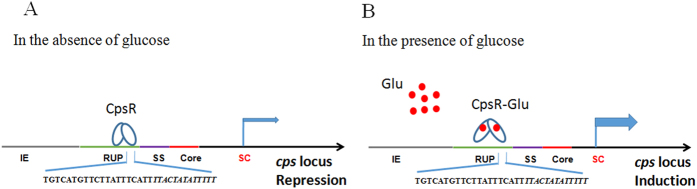
Predicted model for the regulation of the *cps* locus by CpsR in *S. pneumoniae*. In the absence of glucose, the *cps* transcription was inhibited by the CpsR. In the presence of glucose, glucose may lead to conformation changes of CpsR and hence facilitate the transcription of the *cps* locus. IE: insertion element; RUP: repeat sequence of pneumococcus; SS: spacing sequence; Core: core promoter; SC: start codon.

In conclusion, our results provide strong evidence that pneumococci could modulate *cps* gene expression to adapt to changing host environments through CpsR-*cpsp* interaction (Fig. 8). Given the importance of CPS in capsule production and invasive pneumococcal disease, it is rational that the development of small molecular inhibitors targeting the CpsR-mediated encapsulation might be a promising approach against pneumococcal infection.

## Methods

### Bacterial strains and growth conditions

All strains and plasmids are listed in Table S1. Unless stated otherwise, *S. pneumoniae* D39 strain (serotype 2), CMCC31693 strain (serotype 19F) and their derivatives were grown in semi-synthetic casein hydrolysate medium supplemented with 5% yeast extract (C+Y, pH 7.0) medium or blood agar plates at 37 °C under 5% CO_2_. *Escherichia coli* strains were grown in Luria-Bertani (LB) broth with shaking or on LB agar plates at 37 °C. Selective antibiotics were added when necessary.

### DNA affinity purification

Primers were synthesized by Takara Bio, Dalian and listed in Table S2. The forward primer (Pulldown-cps-F) was 5′- labeled with biotin. DNA affinity purification was carried out essentially as described elsewhere^28^. Briefly, the 218-bp biotinylated *cps* promoter fragment (extending from 204 bp upstream of 14 bp downstream of the *cps* locus start codon) was amplified from chromosomal DNA of the *Streptococcus pneumoniae* D39 strain using primers Pulldown-cps-F/R2. The resulting 218-bp DNA fragment was bound to streptavidin-coated magnetic beads (DynabeadsM-280 Streptavidin; Invitrogen, Darmstadt, Germany), followed by incubation with crude extracts obtained from *S. pneumoniae* D39 WT cells grown in the C+Y medium. The eluted proteins were separated by SDS-PAGE, stained with Coomassie, and identified by mass fingerprinting. Nonspecifically bound proteins were removed by several low-salt washing steps (0.1 M NaCl) with subsequent magnetic separation. Specifically bound proteins were eluted with buffers containing 0.3 M, 0.5 M NaCl and subjected to SDS-PAGE and Coomassie staining.

### Electrophoretic mobility-shift assays (EMSA)

The DNA fragments used in electrophoretic mobility shift assays (EMSAs) were generated either by PCR using the corresponding oligonucleotides listed in Table S2 in the supplemental material or by annealing two complementary oligonucleotides by incubating the min an equimolar ratio in annealing buffer (10 mM Tris-HCl [pH 8.0], 50 mM NaCl, 1 mM EDTA) for 5 min at 95 °C, followed by cooling at room temperature. LightShift Chemiluminescent EMSA Kits were purchased from Thermo Scientific. EMSAs were performed according to the manufacturer’s instructions and as described elsewhere with modification^42^. Briefly, EMSA reactions were performed in 20 μl total volumes, containing indicated concentrations of biotinylated DNA probe in buffer containing 50 ng/μl poly (dI–dC). Reaction mixes were incubated for 20 min at room temperature. After incubation, the reaction solution was mixed with 5 μl of the sample loading dye and 20 μl of the solution was subsequently loaded onto pre-cast 6% acrylamide gels (Invitrogen) and electrophoresed at 100 V for 90 min. DNAs were transferred to nylon membranes (Pierce), and cross-linked by exposure to ultraviolet (UV) light. Labeled DNAs were detected using chemiluminescent nucleic acid detection kits (Pierce).

The binding fragment of the 33-mer DNA oligonucleotides was mutated to the indicated sequences, labeled as M6 and M7 (Table S2). Double-stranded DNA were prepared as described elsewhere by annealing complementary oligonucleotides (100 mM each) in 10 mM Tris-HCl, pH 8.0, 20 mM NaCl, heating the reaction to 95 °C for 5 min and allowing it to cool to 25 °C^43^. Wild-type probe or mutated probes (0.25 μM) were incubated with different amounts of purified 6His-CpsR in a 20 μl buffer as that used above for EMSA. In the assays for effects of glucose on the interaction of CpsR and DNA, wild-type probe of 8 fmol was preincubated with 8.0 μg CpsR at room temperature for 15 min before mixing with different concentrations of glucose, followed by the same procedure as in the other assays.

### DNase I footprinting assay

DNase I footprinting assays were performed as described elsewhere by Wang *et al*^44^. Briefly, the *cps* promoter-proximal PCR product was cloned into the HincII digested pUC18H vector (Tolo Biotech). The obtained plasmid was verified via DNA sequencing and used as the template for further preparation of fluorescent FAM labeled probes with primers M13F-47(FAM) and M13R-48. The FAM-labeled probes were purified by the Wizard SV Gel and PCR Clean-Up System (Promega, USA) and were quantified with NanoDrop 2000C (Thermo, USA). For each assay, 400 ng probes were incubated with different amounts of CpsR in a total volume of 40 μl. After incubation for 30 min at 25 °C, 10 μl solution containing about 0.015 unit DNase I (Promega) and 100 nmol freshly prepared CaCl_2_ was added and further incubate for 1 min at 25 °C. The reaction was stopped by adding 140 μl DNase I stop solution (200 mM unbuffered sodium acetate, 30 mM EDTA and 0.15% SDS). Samples were firstly extracted with phenol/chloroform, then precipitated with ethanol and the pellets were dissolved in 30 μl MiniQ water. The preparation of the DNA ladder, electrophoresis and data analysis were the same as described before^44^, except that the GeneScan-LIZ500 size standard (Applied Biosystems) was used.

### Construction of the mutant and complemented (C) Strains

Primers and sequences are listed in Table S2. *cpsR* (*SPD_0064*) gene was deleted from the chromosome of *S. pneumoniae* by insertion-deletion mutagenesis which was performed essentially according to our established protocol^45^. Recombinants were selected on blood agar plates containing erythromycin. Successful replacement by the *erm* cassette was confirmed by sequencing chromosomal DNA in the deleted region.

The full length *cpsR* was amplified from genome DNA of *S. pneumoniae* D39 with primers cpsR-SpeI-F/cpsR-NotI-R (Table S2), respectively. The resulting fragment was cloned into plasmid pJWV25 after digestion with restriction enzymes *Not* I and *Spe* I. A high-fidelity PrimeSTAR DNA polymerase (TaKaRa, Dalian, China) was used to minimize sequence errors during PCR amplification in this study. All PCR amplified sequences were verified by DNA sequencing. The recombinant plasmids were used to transform to obtain the desired strains. All mutations were confirmed by sequencing of chromosomal DNA in the targeted regions.

For pAE03-plasmid based complementation, the fragment including approximately 700 bp upstream of *cps2A* gene was cloned into plasmid pAE03 with *EcoR* I and *Not* I as restriction sites to form *cpsp*^*WT*^*-gfp* transcriptional fusion. For construction of *cpsp*^*M6*^*-gfp* and *cpsp*^*M7*^*-gfp* reporter fusions, *cpsp*^*WT*^*-gfp* fusion was used as template. All primers were listed in Table S2. PCR products generated with *Pfu* polymerase were digested with 1 U *Dpn* I at 37 °C overnight. The resulting products were transformed into *E. coli* DH5α competent cells. Viable mutants were selected on TSA-BA plates supplemented with appropriate antibiotics. Plasmids were used to transform the *S. pneumoniae* D39. The cloned promoter sequences were confirmed by DNA sequencing.

### Overproduction and purification of CpsR protein

The CpsR protein was overproduced in *E. coli* BL21 (DE3) using the expression plasmid pW28-cpsR and purified by nickel chelate affinity chromatography according to the established protocol^46^. Expression was induced at an OD_600_ of 2.0 by addition of 1 mM isopropyl-D-thiogalactoside (IPTG) at 20 °C. Eight hours after induction, cells were harvested by centrifugation, washed once with the appropriate disruption buffer, and disrupted by ultrasonication. The recombinant protein was purified by nickel-nitrilotriacetic acid (Ni-NTA) affinity chromatography (GE Healthcare). Protein concentrations were determined as described above, and the purified proteins were stored at −80 °C until use.

### Immunization of Mice

Mice were immunized as described elsewhere with modification^47^. Briefly, 6His-CpsR antigen was formulated in aluminum hydroxide adjuvant (Alum) to a final ratio of 100 μg of antigen to 1 mg of Alum adjuvant. Balb/c mice were then immunized *i.p*. with three doses of 10 μg at 14-day intervals. Polyclonal mouse serum was collected by cardiac puncture 7 days after the final immunization and stored at 4 °C until use. Mouse antiserum against GADPH was prepared previously in the laboratory by using methods similar to those described for CpsR antigen.

### RNA Extraction and Real-Time RT-PCR

The liquid culture was grown at 37 °C to an OD_600_ = 0.4 determined using a cell density meter (Ultrospec 10, GE Healthcare formerly Amersham Biosciences). Total RNA was immediately stabilized with RNAprotect bacteria reagent (QIAGEN, Valencia, CA) and then extracted from *S. pneumoniae* strains by using a Qiagen RNeasy kit according to the manufacturer’s instructions. RNA was resuspended in nuclease-free water in the presence of RNasin RNase inhibitor (Promega). Contaminating DNA was digested with DNase I (Roche Diagnostics). Real-time RT-PCR was performed on a Rotorgene RG-2000 (Corbett Research, Mortlake NSW, Australia) and as described elsewhere^5^. Briefly, the cDNA (20 ng/reaction) was used for real-time amplification using the primers listed in Table S2. The mRNA level of 16S rRNA was used as an internal control in order to normalize all data. The amounts of transcripts were expressed as the n-fold difference relative to the control gene (2^−ΔCT^, where ΔCT represents the difference in threshold cycle between the target and control genes).

### SDS-PAGE and Western Blotting

Samples were subjected to SDS-PAGE, and separated samples were then electroblotted onto nitrocellulose. After transfer, the membrane was probed with polyclonal murine antisera or antibodies at a dilution of 1:3,000 and then reacted with blotting grade goat anti-mouse IgG alkaline phosphatase conjugate (BioRad). Labeled bands were visualized by Western ECL reagent from Millipore according to the instructions of the manufactures (ChemiDOC XRS+, BIO-RAD).

### Transmission electron microscopy (TEM)

Cells were grown in C+Y medium at 37 °C to the mid-exponential phase. Bacteria were harvested by centrifugation at 4 °C for 10 min at 10,000 × g. Samples were fixed in 2% glutaraldehyde in sodium cacodylate buffer (pH 7.4) for 12 h and then processed by the Electron Microscopy Research Service of Chongqing Medical University. For visualization, cells were sectioned and imaged with a Hitachi H-7500 transmission electron microscope. Image J software was used to determine capsule thickness of 20 randomly chosen bacteria.

### Immunoblotting and ELISA

Pneumococcal CPS was assayed by immunoblotting as described elsewhere with modification^17^. Briefly, one ml of bacterial cultures of the same optical density of 0.4 were pelleted by centrifugation and resuspended in 0.5 ml of DOC solution for 10 min before being used for CPS immunoblotting. The type-2 CPS was detected with a rabbit anti-serotype 2 serum (States Serum Institut, Denmark) and peroxidase-conjugated goat anti-rabbit IgG (H+L) at dilutions of 1: 10000. Binding of the CPS-antibody was visualized by Western ECL reagent from Millipore according to the instructions of the manufactures (ChemiDOC XRS+, BIO-RAD). The results of representative experiments are presented.

### Whole-blood killing assays

Whole-blood-mediated killing assays were carried out as described previously with modification^18^. Bacterial cultures (OD_600_ = 0.4) of the indicated strains were washed twice and resuspended in PBS to generate a suspension of about 1 × 10^5^ CFU/ml. Whole blood from adult Balb/c mice was collected and heparinized. Ten μl bacterial suspensions were mixed with 90 μl heparinized mouse blood and incubated at 37 °C with shaking (180 rpm) for 1, 2, and 3 h, at which time samples were plated on a blood agar plate for enumeration of surviving CFU.

### Animal studies

All animals were purchased from the Laboratory Animal Center of Chongqing Medical University (certificate number: SYXK (yu) 2007-0001). All experimental protocols were approved by the Ethics Committee of Chongqing Medical University. The research was carried out in accordance with the Declaration of Helsinki and with the recommendations in the Guide for the Care and Use of Laboratory Animals of the National Institutes of Health. To investigate the role of CpsR-mediated CPS production in nasopharyngeal colonization, 6- to 8- week old female Balb/c mice were randomly divided into designed groups. Bacteria were grown in C+Y medium to an OD_600_ of approximately 0.4. Mice were anesthetized with pentobarbital sodium (1.5%) prior to challenge by intranasal inoculation with 10^6^ CFU. Bacteria were recovered from mouse nasopharynx at 16 and 24 h, and plated on blood agar plate after appropriate dilutions. For systemic virulence evaluation, 6- to 8- week old female Balb/c mice were intraperitoneally infected with 10^3^ cells of *S. pneumoniae* D39 or their derivates. To evaluate whether the role of CpsR in systemic virulence was influenced by host strain, groups of 10 Balb/c mice were intraperitoneally infected with 10^7^ cells of 31693 strain (serotype 19F) or the Δ*cpsR* mutant, respectively. Mice were monitored at approximately 4-h intervals, and the survival time of each mouse was recorded.

### Statistical analysis

Statistical difference between groups were compared with Student’s t test, the non-parametric Mann-Whitney or Wilcoxon test using GraphPad Prism 5 (GraphPad Software, San Diego, CA). Survival data were analyzed using the log rank (Mantel-Cox) test. Statistical significance was defined as *P* < 0.05.

## Additional Information

**How to cite this article**: Wu, K. *et al*. CpsR, a GntR family regulator, transcriptionally regulates capsular polysaccharide biosynthesis and governs bacterial virulence in *Streptococcus pneumoniae*. *Sci. Rep*. **6**, 29255; doi: 10.1038/srep29255 (2016).

## Supplementary Material

Supplementary Information
