# Supplementary material for: CpsR, a GntR family regulator, transcriptionally regulates capsular polysaccharide biosynthesis and governs bacterial virulence in Streptococcus pneumoniae
Source: Sci Rep. 2016 Jul 8;6:29255. doi: 10.1038/srep29255 (PMC4937376; doi:10.1038/srep29255)
Supplement: Supplementary Information [file srep29255-s1.doc]

| **CpsR, a GntR family regulator, transcriptionally regulates** **capsular polysaccharide biosynthesis and governs bacterial virulence in *Streptococcus pneumoniae***  **Kaifeng Wu^1, *, †^, Hongmei Xu^1, *, &^, Yuqiang Zheng^1^, Libin Wang^1^, Xuemei Zhang^1^, Yibing Yin^1^**   | **Table S1. Manipulated strains and plasmids used in this work.** | | |  | |  | | | --- | --- | --- | --- | --- | --- | --- | | **Strains** | | **Relevant properties** | **Antibiotic Concentrations** | | **Source or reference** | | |  | |  |  | |  | | | D39 | |  |  | | NCTC (National Collection of Type Cultures, London, UK) | | | D39 ΔcpsR (*SPD_0064)* | | Erm^R^, SPD_0064 gene replaced by erm cassette | 0.25 μg/ml | | This study | | | D39 ΔcpsR-compl | | Erm^R^, Tet^R^, containing PczcD-gfp+-CpsR-pJWV25 | Erm: 0.25 μg/ml; Tet: 2.5 μg/ml | | This study | | | *E.coli* BL21(DE3) | | Expression host |  | | Takara | | | CMCC(B)31693 | | S. pneumoniae, serotype 19F, encapsulated |  | | CMCC (National Center for Medical Culture Collections, P. R. China) | | | 19F ΔcpsR (SPD_0064) | | Erm^R^, SPD_0064 gene replaced by erm cassette | 0.25 μg/ml | | This study | | | D39 cpsp^WT^ | | D39 derivative, contaning structure cpsp^WT^-gfp+-pAE03, Erm^R^ | 0.25 μg/ml | | This study | | | D39 cpspM6 | | D39 derivative, contaning structure cpsp^M6^-gfp+-pAE03, Erm^R^ | 0.25 μg/ml | | This study | | | D39 cpspM7 | | D39 derivative, contaning structure cpsp^M7^-gfp+-pAE03, Erm^R^ | 0.25 μg/ml | | This study | | |  | |  |  | |  | | | **Plasmids** | | | | | | | | pJWV25 | Tet^R^ | | |  | | [Veening, et al. (2009)](../../../../C:%5CUsers%5CWuk%5CAppData%5CRoaming%5CMicrosoft%5CExcel%5C1672%20Pulldown%20proteins%20(version%201).xlsb#Sheet1!_ENREF_2) | | pEVP3 | Chl^R^ | | |  | | Pestova and Morrison (1998) | | pYYB19 | pW28, cpsR, kana^R^ | | |  | | This study | | pYYB20 | pJWV25, CpsR, Tet^R^ | | |  | | This study | | PYYB21 | pAE03, cpsp^WT^, Erm^R^ | | |  | | This study | | pYYB27 | pAE03, cpsp^M6^, Erm^R^ | | |  | | This study | | pYYB28 | pAE03, cpsp^M7^, Erm^R^ | | |  | | This study | | **Reference** |  | | |  | |  | | 1 | **Pestova EV, Morrison DA.** 1998. Isolation and characterization of three Streptococcus pneumoniae transformation-specific loci by use of a lacZ reporter insertion vector. J Bacteriol. **180 (10):** 2701-10. | | |  | |  | |  | **Eberhardt A, Wu LJ, Errington J, Vollmer W, Veening JW.** 2009. Cellular localization of choline-utilization proteins in Streptococcus pneumoniae using novel fluorescent reporter systems. Mol Microbiol. **74:**395-408. | | |  | |  | |
| --- | --- | --- | --- | --- | --- | --- | --- | --- | --- | --- | --- | --- | --- | --- | --- | --- | --- | --- | --- | --- | --- | --- | --- | --- | --- | --- | --- | --- | --- | --- | --- | --- | --- | --- | --- | --- | --- | --- | --- | --- | --- | --- | --- | --- | --- | --- | --- | --- | --- | --- | --- | --- | --- | --- | --- | --- | --- | --- | --- | --- | --- | --- | --- | --- | --- | --- | --- | --- | --- | --- | --- | --- | --- | --- | --- | --- | --- | --- | --- | --- | --- | --- | --- | --- | --- | --- | --- | --- | --- | --- | --- | --- | --- | --- | --- | --- | --- | --- | --- | --- | --- | --- | --- | --- | --- | --- | --- | --- | --- | --- | --- | --- | --- | --- | --- | --- | --- | --- | --- | --- | --- | --- | --- | --- | --- | --- | --- | --- | --- | --- | --- | --- | --- | --- | --- | --- | --- | --- | --- | --- | --- | --- | --- | --- | --- | --- | --- | --- | --- | --- | --- | --- | --- | --- | --- | --- | --- | --- | --- | --- | --- | --- | --- | --- | --- | --- | --- | --- |

| Table S2. Primers or oligonucleotides used in this study. | |
| --- | --- |
| **CPS Promter region** | Range: 313543 to 313760 (218 bp) |
| **Length of the amplified fragment** | 218 bp (including a 14-bp fragment downstream of the start codon ATG) |
| **Sequence of the Pulldown probe (5'-botin labeled)** | TACACATCTGCTTCTAAAATATTGTTAGAAAACGATTTGACTGTCCTGATCAATTTGTCATGTTCTTATTTCATTTTACTATATTTTTGGTTCGCGGGAAGTCTACTAAGATACTTAAAGATGCAGATAGTGAAAAAAGGTGTAGACATTACCGTAAAAAAGTGATATAATCGTAAGATGTTCAATGTATAGGTGTTAATCATGAGTAGACGTTTTAA |
|  |  |
| **Primers used for Pulldown** | Sequence (5'-3') |
| Pulldown -cps-F | Biotin-TACACATCTGCTTCTAAAATATTGT |
| Pulldown -cps-R2 | TTAAAACGTCTACTCATGATTAACA |
| Primers used for construction SPD_0064 deletion mutant | |
| SPD_0064 UP P1 | TCCAAGCAACATAAGTCTCTACCGT |
| SPD_0064 UP P2 | ATCAAACAAATTTTGGGCCCGGAGAAAATTTTACCATAAAAGCGAA |
| SPD_0064 DW P3 | ATTCTATGAGTCGCTGCCGACTCTAAGAAGAAAGCCTGAGCCTAATC |
| SPD_0064 DW P4 | TCTTCTTCCTTTTTATTAGCAGTTT |
| erm F | CCGGGCCCAAAATTTGTTTGAT |
| erm R | AGTCGGCAGCGACTCATAGAAT |
| Primers used for expression of 6His-tagged CpsR | |
| cpsR-NdeI-F | GGAATTCCATATGGAGAAGGAAAATAGAGGT |
| cpsR-XhoI-R | CCGCTCGAGTTAGTGATCACGGTCACATGAG |
| for complementation with plasmid pJWV25 | |
| cpsR-SpeI-F | GGACTAGTATGGAGAAGGAAAATAGAGG |
| cpsR-NotI-R | ATAAGAATGCGGCCGCTTAGTGATCACGGTCACATG |
| for gfp-fusion reporter |  |
| F-cpsp^WT^-pae03 | CCGGAATTCAAGAAATCCTCTGATATCTTC |
| R-cpsp^WT^-pae03 | ATAAGAATGCGGCCGCACATGATTAACACCTATACATTGAAC |
| F-cpsp^M6^-pae03 | TGTCATGTTCCCGCCTCATTTTACTGCGCCTTT |
| R-cpspM6-pae03 | AAAGGCGCAGTAAAATGAGGCGGGAACATGACA |
| F-cpsp^M7^-pae03 | TGTCGCACCCTTATTTCGCCCCACTATATTTTT |
| R-cpsp^M7^-pae03 | AAAAATATAGTGGGGCGAAATAAGGGTGCGACA |
| oligonucleotides |  |
| WT F | 5’-biotin-TGTCATGTTCTTATTTCATTTTACTATATTTTT |
| WT R | 5’ AAAAATATAGTAAAATGAAATAAGAACATGACA |
| M6 F | 5’-biotin-TGTCATGTTCCCGCCTCATTTTACTGCGCCTTT |
| M6 R | 5’ AAAGGCGCAGTAAAATGAGGCGGGAACATGACA |
| M7 F | 5'-biotin-TGTCGCACCCTTATTTCGCCCCACTATATTTTT |
| M7 R | 5’ AAAAATATAGTGGGGCGAAATAAGGGTGCGACA |
| Primers used in qRT-PCR |  |
| 16s rRNA-F | GTAGTCCACGCTGAAACGATGATG |
| 16s rRNA-R | CTGTCCCGAAGGAAAACTCTATCT |
| *cps2A-F* | CGTCAACCGAAGCACTG |
| cps2A-R | GATCCATCCGACCTGTCC |
| *cps2B-F* | TCGTTATGCCTTGATAGAAT |
| cps2B-R | ATTTACTTGCGTGTAACAGC  TTTGCAGGCAGGATCTTATC |
| *cps2C-F* |
| cps2C-R | GGCTTCCTCTGGCTGTTTAT |

Note: The underlines denote the reverse complemented sequences of Erm F/R.


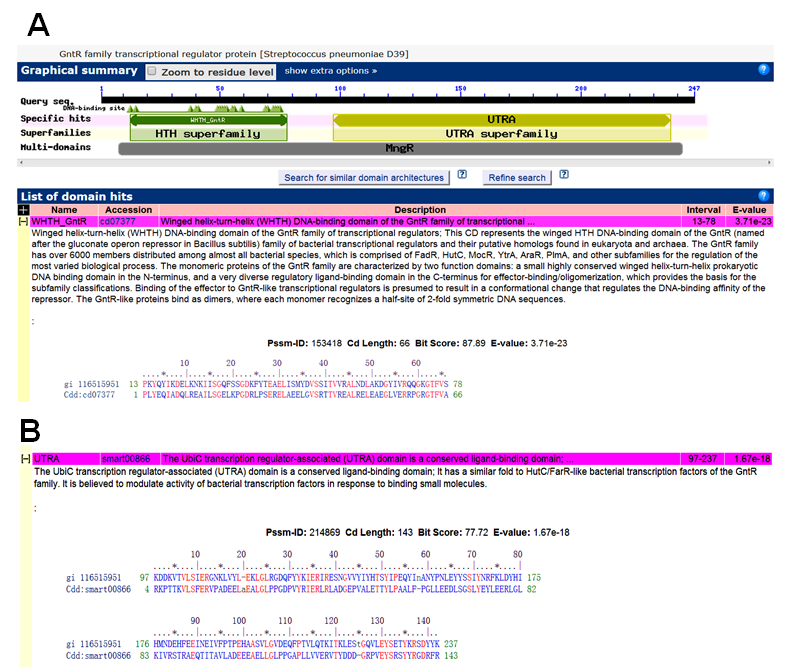


**Figure S1. Conserved domains of CpsR**


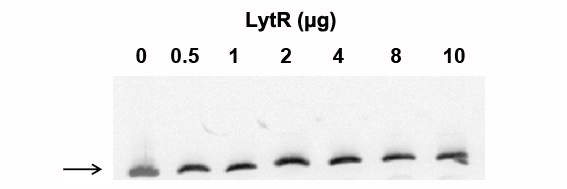


**Figure S2. The binding property of LytR (SPD_1741) with the *cpsp*.**

**The arrow indicates the unbound probe.**


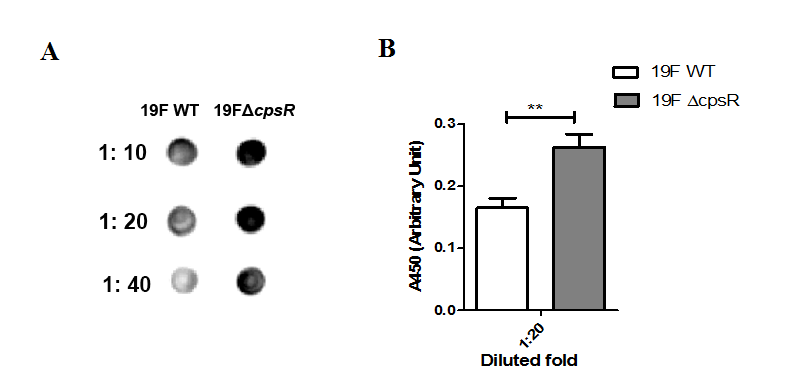


**Figure S3. Detection of CPS production in serotype 19F and Δ*cpsR* by dot blot. (**A) Image of a representative immunodot blot from at least three independent experiments showing a 2-fold dilution series with an initial dilution of 1:10 for the wild type and Δ*cpsR* mutant strains. The numbers on the left side indicate the dilutions. (B) CPS levels were determined by ELISA. Data represent mean + SD from three independent experiments. **, P < 0.01.
